# A trypanosomal orthologue of an intermembrane space chaperone has a non-canonical function in biogenesis of the single mitochondrial inner membrane protein translocase

**DOI:** 10.1371/journal.ppat.1006550

**Published:** 2017-08-21

**Authors:** Christoph Wenger, Silke Oeljeklaus, Bettina Warscheid, André Schneider, Anke Harsman

**Affiliations:** 1 Department of Chemistry and Biochemistry, University of Bern, Freiestrasse 3, Bern, Switzerland; 2 Department of Biochemistry and Functional Proteomics, Institute of Biology II, Faculty of Biology, University of Freiburg, Schänzlestr. 1, Freiburg, Germany; 3 BIOSS Centre for Biological Signalling Studies, University of Freiburg, Schänzlestr. 18, Freiburg, Germany; University of California, Los Angeles, UNITED STATES

## Abstract

Mitochondrial protein import is essential for *Trypanosoma brucei* across its life cycle and mediated by membrane-embedded heterooligomeric protein complexes, which mainly consist of trypanosomatid-specific subunits. However, trypanosomes contain orthologues of small Tim chaperones that escort hydrophobic proteins across the intermembrane space. Here we have experimentally analyzed three novel trypanosomal small Tim proteins, one of which contains only an incomplete Cx3C motif. RNAi-mediated ablation of TbERV1 shows that their import, as in other organisms, depends on the MIA pathway. Submitochondrial fractionation combined with immunoprecipitation and BN-PAGE reveals two pools of small Tim proteins: a soluble fraction forming 70 kDa complexes, consistent with hexamers and a second fraction that is tightly associated with the single trypanosomal TIM complex. RNAi-mediated ablation of the three proteins leads to a growth arrest and inhibits the formation of the TIM complex. In line with these findings, the changes in the mitochondrial proteome induced by ablation of one small Tim phenocopy the effects observed after ablation of TbTim17. Thus, the trypanosomal small Tims play an unexpected and essential role in the biogenesis of the single TIM complex, which for one of them is not linked to import of TbTim17.

## Author summary

*Trypanosoma brucei* and its relatives are prominent pathogens causing human and animal diseases, which mainly affect developing countries. The single mitochondrion of trypanosomes is essential across its entire life cycle. Most organellar proteins are imported by hetero-oligomeric protein complexes in the two mitochondrial membranes. Interestingly, the composition of the two import machineries is remarkably different from their corresponding counterparts in other organisms. In contrast, chaperones termed small Tims that escort hydrophobic proteins across the aqueous intermembrane space are conserved in almost all eukaryotes including trypanosomes. Here we show that a fraction of them interact tightly with the inner membrane translocase. Another fraction is present as soluble 70 kDa complexes, which likely consists of hexamers of small Tims without a defined subunit composition. In other eukaryotes these hexamers are usually composed of two alternating small Tims. Moreover, while some small Tims are involved in import of a core subunit of the inner membrane protein translocase, we found one small Tim that directly mediates the assembly of the translocase complex. Knowing which components of the trypanosomal protein import systems are conserved and which ones are not is essential to evaluate whether mitochondrial protein import might be a suitable drug target.

## Introduction

The parasitic protozoan *Trypanosoma brucei* is the causative agent of the devastating human sleeping sickness and of nagana in cattle [1]. However, besides its clinical and economic importance, *T*. *brucei* is also an interesting model to investigate variations of basic cell biological processes [2, 3]. One such process is mitochondrial protein import, which has been studied in great detail in *Saccharomyces cerevisiae* and in mammalian cells [4, 5]. Modern phylogeny divides eukaryotes into five to six supergroups that diverged very early in evolution [6]. Fungi and animals belong to the supergroup of the Opisthokonts and therefore are quite closely related. Trypanosomes are a member of the supergroup of the Excavates and thus are phylogenetically very distant to Opisthokonts [7]. Due to this position in the eukaryotic evolutionary tree and its experimental accessibility, *T*. *brucei* is excellently suited to investigate which features of mitochondrial protein import are conserved and which ones are not [3, 4]. Recent studies in *T*. *brucei* have characterized the main protein translocase of the mitochondrial outer membrane (TOM), termed archaic translocase of the OM (ATOM) [8, 9], as well as the translocase of the inner membrane (TIM) [10]. Only two subunits of the ATOM complex and one integral membrane subunit of the single trypanosomal TIM complex are orthologous to TOM and TIM complex subunits of any other eukaryote [3, 4]. This is surprising since protein import is considered to be one of the first, if not the first, mitochondria-specific trait to evolve, which suggests that the machineries mediating the process would be conserved.

In the present study we focus on the small Tim family of intermembrane space (IMS) localized chaperones (also known as tiny Tims), which is conserved in all mitochondria-containing eukaryotes including trypanosomes [11, 12]. Members of the small Tim family have a molecular weight of around 10 kDa and contain conserved twin Cx3C motifs that normally are separated by 11–16 residues [13]. The two motifs form intramolecular disulfide bonds that stabilize the helix-loop-helix structure of small Tim proteins [14, 15]. Their function in yeast and humans is to guide hydrophobic import substrates that emerge from the TOM complex across the IMS to the respective insertases [13]. Mitochondrial carrier proteins (MCPs), which usually contain 6 transmembrane spanning domains, and other inner membrane (IM) proteins with internal targeting signals are transferred by small Tims to the TIM22 complex in the IM [16, 17]. Similarly, β-barrel proteins of the OM are handed over to the sorting and assembly machinery (SAM) [18, 19]. To this end, small Tim proteins form ring-like hetero-hexameric oligomers. In yeast, two such structures have been characterized consisting of either alternating Tim9/Tim10 or Tim8/Tim13 subunits [14, 20, 21]. Moreover, a small fraction of Tim9/Tim10 is associated with Tim12 and binds to the TIM22 complex [22]. Similarly, in human mitochondria, two hexameric complexes consisting of Tim9/Tim10a or DDP1 (deafness dystonia peptide 1)/Tim13 as well as a Tim9/Tim10a/Tim10b complex that associates with TIM22 can be differentiated [23, 24].

Like most mitochondrial proteins the small Tims are imported. Their import, similar to that of other small cysteine-rich IMS proteins, is coupled to oxidation of their cysteine residues [25]. Small Tim proteins are first translocated across the OM through the TOM complex. Subsequently they engage with Mia40 of the mitochondrial IMS import and assembly machinery (MIA), which in cooperation with the sulfhydryl oxidase Erv1 promotes oxidation and folding of the proteins before they assemble into the hexameric complexes or associate with the TIM22 complex [26–28].

Bioinformatic analysis predicts that the *T*. *brucei* genome encodes five classical small Tims, as well as one small Tim-like protein that only contains two cysteine residues instead of the classical twin Cx3C motif [3, 10–12]. Merely a single one of these proteins has been experimentally analyzed to a very limited extent [11]. Here we present the first detailed experimental analysis of trypanosomal small Tim proteins. It focuses on the three most recently discovered proteins, which includes the atypical small Tim. We demonstrate that mitochondrial import of these proteins depends on the trypanosomal TbERV1 orthologue. Moreover, we show that the three small Tims are subunits of the single trypanosomal TIM complex (approximately 700 kDa) [10], but are also present in soluble complexes of approximately 70 kDa. Ablation of any of the three proteins inhibits normal growth, results in the disappearance of the TIM complex and causes mitochondrial protein import defects. While individual ablation of two of the proteins affects import of TbTim17, a core subunit of the single trypanosomal TIM complex, we provide evidence that one small Tim protein is directly involved in TIM complex assembly.

## Results

### Three novel trypanosomal small Tims

**Fig 1:**
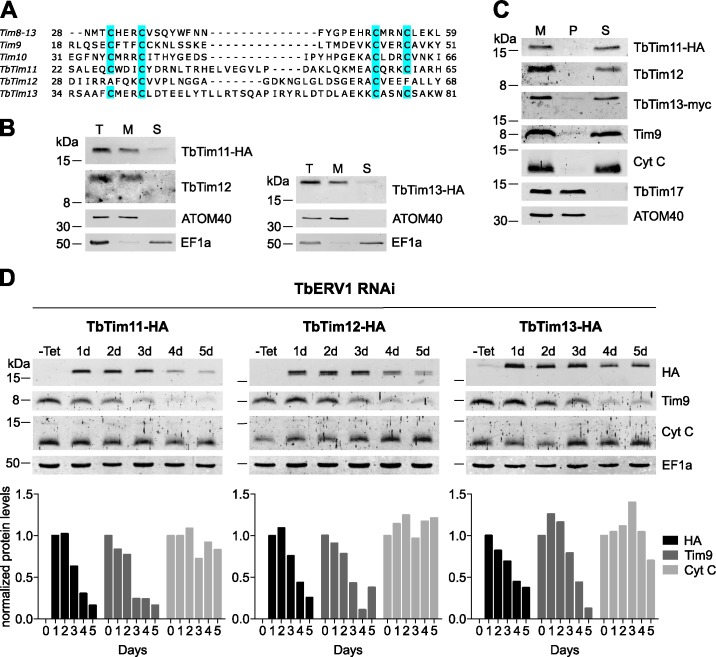
TbTim11, TbTim12 and TbTim13 are novel trypanosomal members of the small Tim family. A) Sequence alignment of putative novel small Tims with known small Tims from trypanosomal mitochondria demonstrates conservation of twin CX3C motifs in all candidates except TbTim12. B) Total cells (T) were treated with 0.015% digitonin to separate a mitochondria enriched fraction (M) from the cytosol-containing supernatant (S). The tagged and endogenous small Tim-like proteins co-fractionate with the mitochondrial marker ATOM40, while the cytosolic protein elongation factor 1A (EF1a) stays in the cytosol. C) Alkaline carbonate extraction at pH 11.5 was performed on digitonin extracted crude mitochondria (M). The resulting pellet fraction (P) contains mitochondrial membrane proteins such as TbTim17 and ATOM40, while the soluble marker protein cytochrome c (Cyt C) is released to the supernatant (S). For this experiment, a cell line co-expressing TbTim11-HA and TbTim13-myc was used. D) Inducible expression of individual tagged small Tim candidates in the background of TbERV1 RNAi. Steady state levels of tagged candidates and endogenous small Tim9 are analyzed by immunoblotting. Cyt C is an IMS protein whose import is independent of the MIA pathway [30] and the cytosolic protein EF1a serves as loading control. The lower panel depicts a densitometric quantification of the western blot results. Values were normalized to those of EF1a. For a characterization of the three ERV1-RNAi cell lines see S1 Fig.

Using bioinformatics, Gentle et al. detected three trypanosomal small Tim proteins, termed Tim9, Tim10 and Tim8-13 [11]. In a more recent compositional characterization of the single trypanosomal TIM complex, three additional such proteins were discovered. Based on their molecular weight they were termed TbTim11 (Tb927.5.3340), TbTim12 (Tb927.4.3430) and TbTim13 (Tb927.10.11520). The characteristic twin Cx3C motifs that stabilize the hairpin structure of small Tims and target them to the MIA pathway in other eukaryotes are conserved in five of these trypanosomal small Tims [11] (Fig 1A). TbTim12 however contains only two cysteines that are separated by 22 amino acids, indicating a lack of the outer disulfide bond [12]. This is a unique feature not found in any other small Tim so far. Sequence comparison of trypanosomal small Tims with their counterparts in yeast and human indicates that it is not possible to assign clear yeast or human orthologues to the newly identified trypanosomal small Tims (S1 Table). It has been shown that all trypanosomal small Tims are associated with the single trypanosomal TIM complex, irrespectively of whether it is engaged in MCP import or in import of presequence containing proteins [10]. The latter is unexpected as presequence-containing proteins do not require small Tims to cross the IMS.

In order to determine the intracellular localization of the three newly discovered small Tim proteins, we established cell lines allowing inducible ectopic expression of C-terminally myc- and HA-tagged versions of the three proteins in various combinations. Cell lines expressing tagged TbTim11 and TbTim13 were subjected to digitonin-based cell fractionation. The results in Fig 1B show that both tagged proteins as well as the endogenous TbTim12 co-fractionated with the mitochondrial marker ATOM40. Furthermore, alkaline carbonate extraction at pH 11.5 of such crude mitochondrial fractions demonstrated that all three proteins are soluble proteins and behave identical to the IMS-localized peripheral membrane protein cytochrome C (Fig 1C). Import of small Tim proteins into the IMS of yeast and human mitochondria depends on the MIA pathway. We therefore expect the same to be the case for the trypanosomal proteins. To test this prediction we expressed the tagged versions of TbTim11, TbTim12 and TbTim13 individually in the background of an RNAi cell line allowing for ablation of TbERV1, the only known component of the trypanosomal MIA pathway [29]. We have previously shown that inhibition of protein import by ablation of import factors results in rapid degradation of the corresponding non-imported substrate proteins in the cytosol [9]. In line with this, Fig 1D shows that the levels of all three novel small Tim proteins were drastically reduced upon TbERV1 RNAi induction. The same was true for endogenous Tim9, but not for cytochrome C, which is imported in a MIA-independent fashion [30]. This indicates that TbTim11, TbTim13 as well as TbTim12 are substrates of the trypanosomal MIA pathway consistent with a localization in the mitochondrial IMS.

### Trypanosomal small Tims form soluble and membrane associated complexes

**Fig 2:**
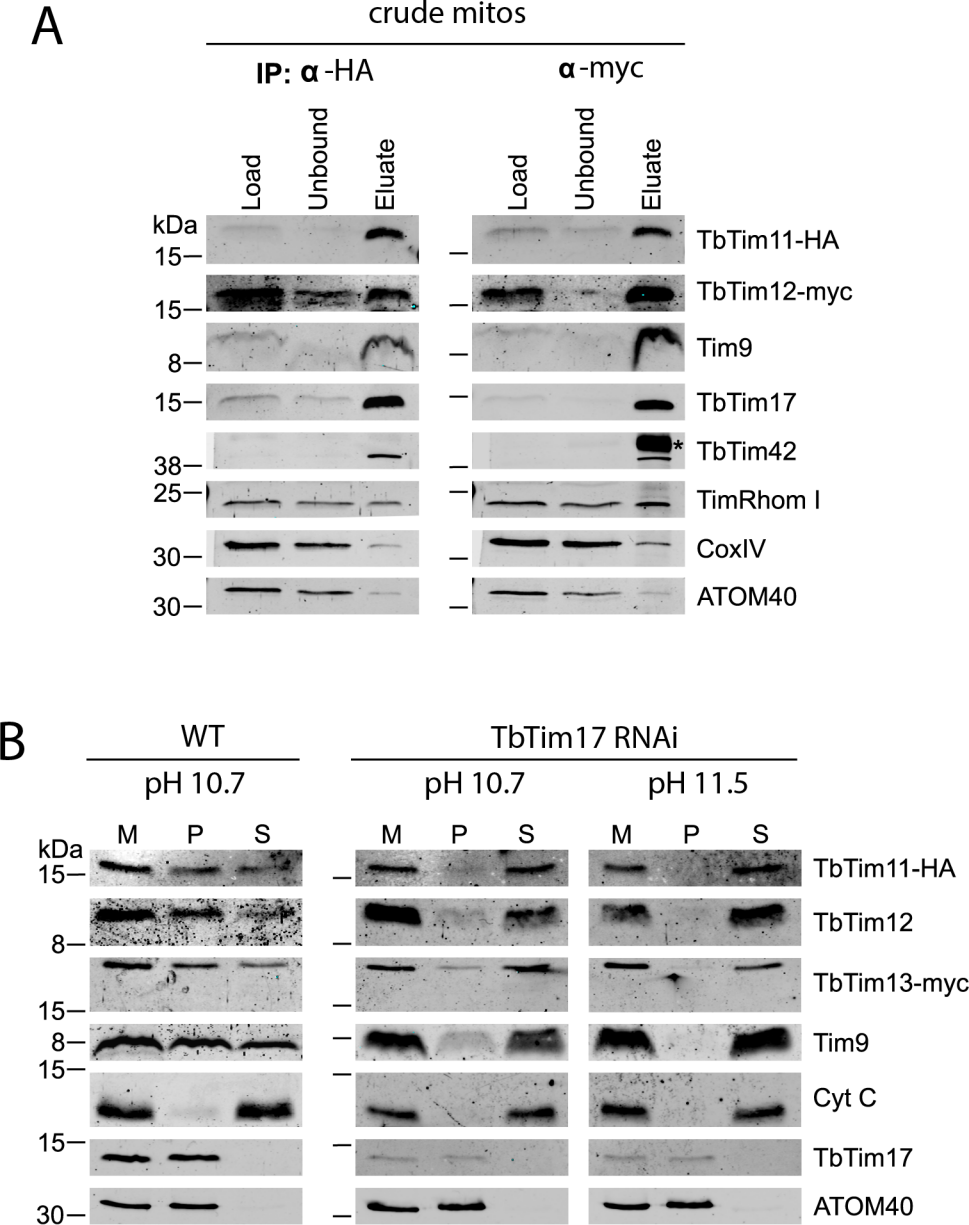
Small Tims are bound to the IM by association with the TIM complex. A) A cell line co-expressing TbTim11-HA and TbTim12-myc was subjected to co-immunoprecipitation targeting either the HA- (left panel) or the myc-tagged protein (right panel). 5% of the respective lysate (”Load”), 5% of the unbound proteins after IP (“Unbound”) and 100% of the final eluate (“Eluate”) were subjected to SDS-PAGE and western blotting. The blots were probed for the tagged small Tims and the TIM components Tim9, TbTim17, TbTim42 and TimRhom I. ATOM40, the central components of the OM translocase, and the cytochrome oxidase subunit IV (CoxIV) served as controls. The asterisk denotes the co-eluted heavy chain of the anti-myc antibody. B) Alkaline carbonate extraction at low (pH 10.7) and high stringency (pH 11.5) was performed on digitonin-extracted crude mitochondria (M). Mitochondrial transmembrane proteins such as TbTim17 and ATOM40 as well as tightly associated proteins are retained in the resulting pellet fraction (P), while the soluble marker protein cytochrome c (Cyt C) is released to the supernatant (S). Cell lines co-expressing TbTim11-HA and TbTim13-myc in either wildtype (WT) or TbTim17 RNAi background (2 days induced) were used to analyze small Tim fractionation in the presence (left panel) or absence of TbTim17 (right panel).

The initial identification of the novel small Tims was based on a set of reciprocal co-immunoprecipitations targeting TbTim17 and TbTim13. In these experiments all six trypanosomal small Tims were specifically interacting with both bait proteins [10]. This stands in contrast to other organisms in which some small Tims exclusively form soluble complexes. Thus, we extended our analysis and performed pulldowns of crude mitochondrial fractions (termed "crude mito") with TbTim11-HA and TbTim12-myc (Fig 2A). The results show that the two tagged proteins, the endogenous Tim9 as well as the integral membrane TIM subunits TbTim17, TbTim42 and TimRhom I are enriched in the eluate, whereas CoxIV and ATOM40 essentially remain in the unbound fractions. In summary, these results confirm the reciprocal interaction of the trypanosomal small Tims and their association with the single TIM complex.

Even though alkaline extraction at pH 11.5 had demonstrated that the novel small Tims are soluble proteins (Fig 1C), the interaction with transmembrane components of the TIM complex suggests an association with the mitochondrial inner membrane. In order to analyze this further, we subjected a cell line expressing TbTim11-HA and TbTim13-myc to alkaline extraction at pH 10.7. Under these less stringent conditions, all detectable small Tims were partially found in the insoluble pellet fraction, while the peripheral membrane protein cytochrome C was still exclusively detected in the soluble fraction (Fig 2B). However, if the experiment was repeated in a cell line ablated for TbTim17, all analyzed small Tims (TbTim11, TbTim12, TbTim13 and Tim9) were exclusively detected in the soluble fraction, regardless of the extraction conditions used (Fig 2B). These results confirm that the partial association of small Tims with the mitochondrial IM depends on the presence of the TIM complex. Moreover, they reveal a strong interaction between the small Tims and the integral membrane subunits of the TIM complex that, unlike most typical interactions between peripheral and integral membrane proteins, is in part resistant to highly alkaline conditions.

**Fig 3:**
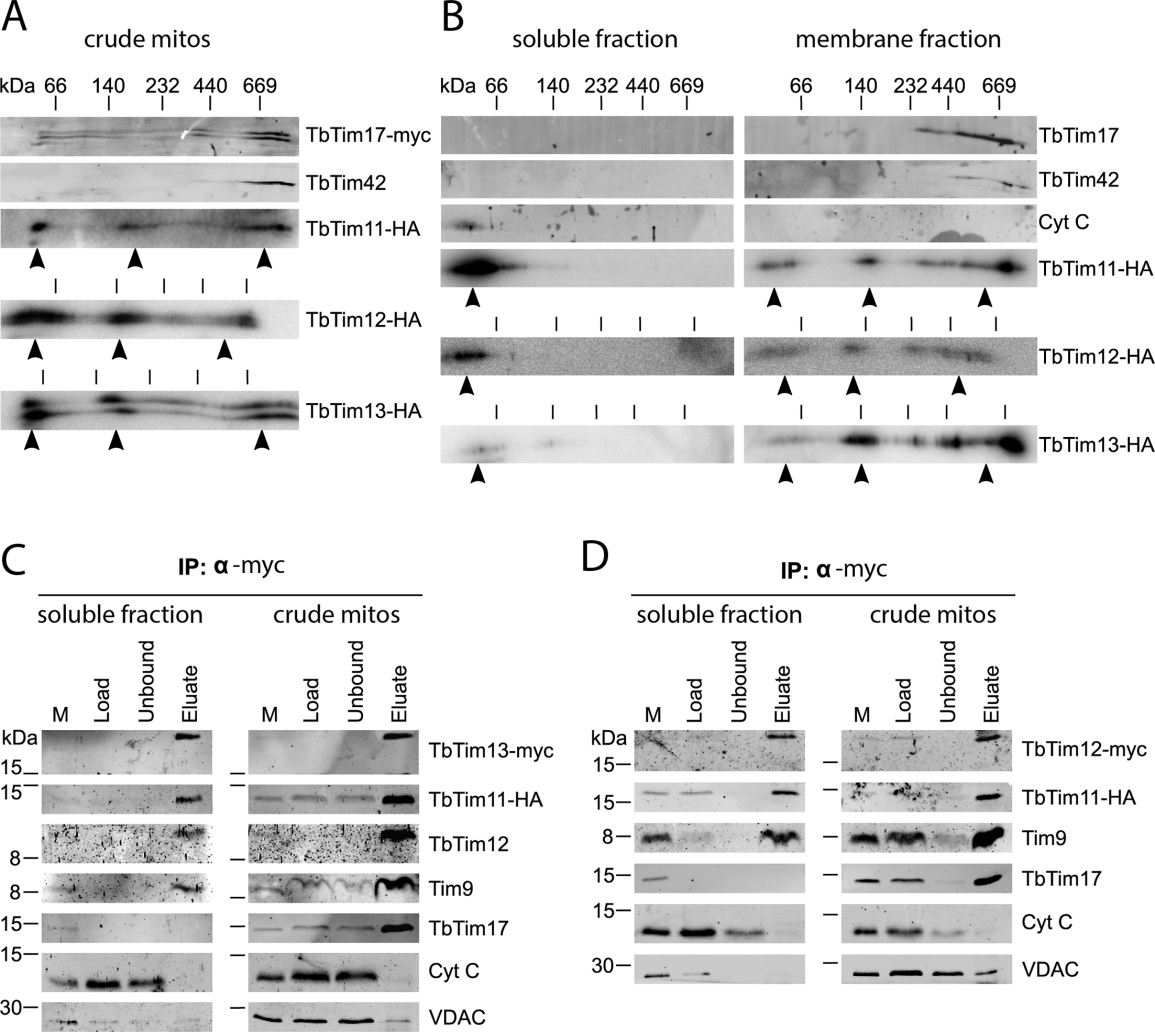
Small Tims form soluble complexes of approximately 70 kDa. A) 2D BN-PAGE analysis of digitonin solubilized crude mitochondrial fractions. Lysates of cell lines expressing TbTim17-myc and one of the HA-tagged novel small Tims were combined and subjected to 6–16.5% BN PAGE in the first dimension, followed by 14% SDS PAGE in the second dimension and finally western blotting. The gels were aligned to the 66 kDa marker. The TIM complex components TbTim17-myc and TbTim42 were detected in all three analyses along with the respective HA-tagged small Tims. The control blots for the analyses of TbTim11-HA and TbTim13-HA can be found in S2 Fig. Arrowheads indicate the approximate positions of the high molecular weight complexes containing small Tim proteins. B) 2D BN-PAGE analysis of submitochondrial fractions. The soluble content of the IMS (“soluble fraction”) was separated from the “membrane fraction” using 0.1% digitonin. Solubilized proteins were separately subjected to 2D-BN/SDS-PAGE as described above. Cyt C serves as a marker for soluble IMS proteins. Arrowheads indicate high molecular weight complexes containing small Tim proteins. C) Co-immunoprecipitation from submitochondrial fractions targeting myc-tagged TbTim13. A cell line co-expressing TbTim11-HA and TbTim13-myc was used to prepare a crude mitochondrial fraction or a “soluble fraction” containing IMS proteins by differential digitonin extraction. 5% of the initial crude mitochondrial fraction (M) and the same amounts of “Load”, “Unbound” and “Eluate” as in Fig 2A were separated on SDS-PAGE and subjected to western blotting. The TIM complex component TbTim17 as well as the small Tims Tim9 and TbTim12 were detected by specific antibodies along with the HA- and myc-tagged other small Tims. The IMS protein Cyt C and the outer membrane protein VDAC were detected to confirm proper fractionation. D) Same experiment as in (C) targeting myc-tagged TbTim12 in a cell line co-expressing TbTim11-HA.

To assess the organization of the small Tim-containing complexes further, we performed 2D-blue native (BN)/SDS-PAGE analysis using crude mitochondrial fractions solubilized with 1% digitonin (Fig 3A). The three novel small Tims were found in a heterogenous population of protein complexes of approximately 70 kDa, 150 kDa and ≥700 kDa in size. While most of the high molecular weight complexes co-migrate with the ones formed by the transmembrane subunits TbTim17 and TbTim42 of the TIM complex, the smallest complexes might correspond to the soluble 70 kDa hetero-hexameric complexes formed by small Tims in yeast and humans. To assess if any of these complexes are soluble, we repeated the 2D BN-PAGE using mitochondrial subfractions. To that end, we lysed the OM of crude mitochondria using 0.2% digitonin. The released soluble fraction contains proteins of the IMS (termed "soluble fraction"), whereas the remaining pellet fraction consists mainly of IM proteins (termed "membrane fraction"). The results in Fig 3B show that the only small Tim-containing complexes present in the soluble fraction are approximately 70 kDa in size, whereas the higher molecular weight complexes were exclusively found in the membrane fraction. The uniform size of the soluble small Tim complexes of approximately 70 kDa suggests the existence of hexameric assemblies.

### Composition of small Tim complexes

Next, we separately analyzed the composition of the soluble and membrane-associated complexes formed by the novel small Tims by co-immunoprecipitations based on the submitochondrial fractions described above. Combination cell lines expressing two differently tagged small Tims and the use of peptide antibodies against endogenous Tim9 and TbTim12 allowed us to probe for up to 4 small Tims in a single pulldown experiment. The results show that in pulldowns of the soluble fraction all detectable small Tims co-precipitate with either TbTim13 (Fig 3C) or TbTim12 (Fig 3D). The only difference observed in pulldowns of the respective solubilized crude mitochondria was that also TbTim17 was recovered. This demonstrates that there are at least two major populations of trypanosomal small Tim complexes, soluble ones in the IMS which contain all small Tims but no other TIM complex subunits, and another one in which all small Tims are associated with the TIM complex of the inner membrane [10].

**Fig 4:**
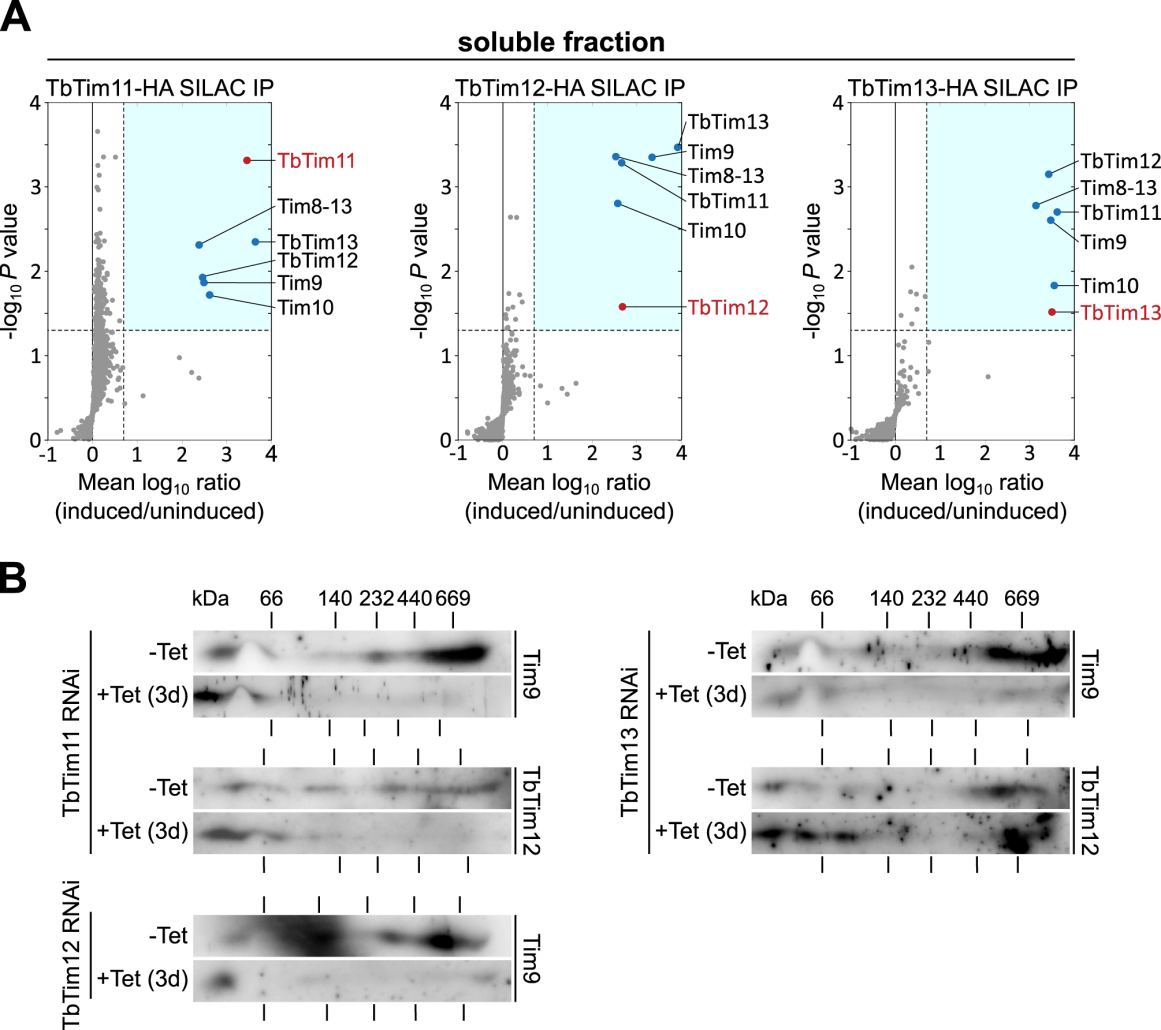
Composition of soluble small Tim complexes. A) Quantitative MS analysis of SILAC-immunoprecipitations of the indicated HA-tagged small Tim protein from IMS protein-containing “soluble fractions” that were produced by differential digitonin extraction. Mean log_10_ ratios (induced/uninduced) of proteins detected by quantitative MS in ≥ two out of three independent experiments are plotted against the corresponding–log_10_ *P* values (one-sided *t* test). Horizontal dashed lines indicate a *P* value of 0.05, whereas the vertical black dashed lines mark a fivefold enrichment. The bait proteins are indicated in red. For a complete list of proteins, see S2 Table. B) 2D BN-PAGE analysis of crude mitochondrial fractions of uninduced and induced (3 days) TbTim11, TbTim12 and TbTim13-RNAi cell lines. Immunoblots were probed for native untagged Tim9 and TbTim12 (for the TbTim11 and TbTim13-RNAi cell lines) and for Tim9 (for the TbTim12-RNAi cell line). Since untagged small Tim proteins were detected the molecular weight of the putative small Tim hexamers is lower than in Fig 3A and 3B.

To analyze the composition of the soluble small TIM complexes in more detail, we performed SILAC-based co-immunoprecipitation experiments using the *T*. *brucei* cell lines allowing inducible, ectopic expression of C-terminally HA-tagged versions of TbTim11, TbTim12 and TbTim13, respectively. Each of these cell lines was grown in the presence or absence of tetracycline and isotopically-labeled heavy or light lysine and arginine respectively. Subsequently, identical cell numbers of both populations were mixed, crude mitochondria were prepared and their OM was lysed with 0.2% of digitonin. The resulting fractions containing the soluble small Tim-containing complexes were subjected to immunoprecipitations using anti-HA antibodies and analyzed by quantitative MS. The results yield a consistent picture: in all three cases all six small Tims were highly enriched in the IPs and no other significantly enriched proteins were detected (Fig 4A) (S2 Table).

The molecular weight of the small Tim-containing complexes of close to 70 kDa suggests they are present in hexameric assemblies. However, the uniform recovery of all six small Tims in all pull down experiments excludes that the postulated hexamers, as in yeast and mammals, are formed by specific pairs of small Tims. Two alternative quaternary structures that are consistent with the experimental evidence would be: i) that all six small Tims build a single defined hexamer consisting of six different subunits, or ii) that the six different small Tim subunits form promiscuous hexamers without defined subunit compositions. Should a single defined hexamer exist we would expect that ablation of any of the six small Tim subunits would result in the collapse of the hexamer. However, in the case of heterogeneous hexamers ablation of a single Tim subunit would not affect hexamer formation as its absence would be compensated for by the other members of the small Tim protein family. 2D BN-PAGE analysis of the soluble complexes in uninduced and induced TbTim11, TbTim12 and TbTim13-RNAi cell lines favours the second model, since ablation of the corresponding small Tims does not significantly affect the soluble complexes containing other small Tim proteins that were not targeted by the RNAi (Fig 4B).

### TbTim13 is involved in TIM complex biogenesis

**Fig 5:**
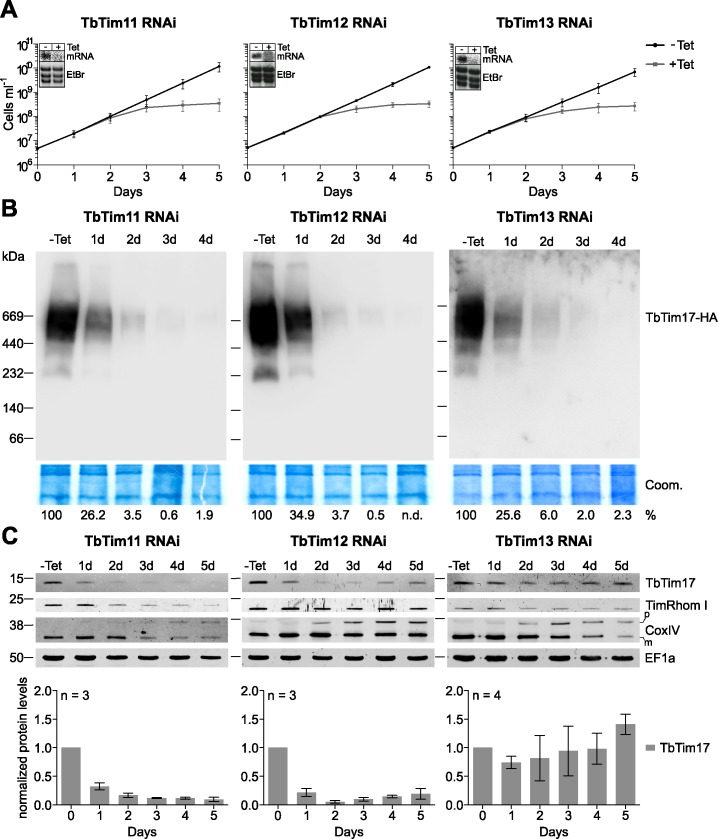
Novel small Tims are essential for TIM complex biogenesis. A) Growth curves of uninduced (-Tet) and induced (+Tet) procyclic RNAi cell lines ablating either, TbTim11, TbTim12 or TbTim13. The insets show Northern blots of total RNA extracts of uninduced (-Tet) and 2 days induced cells (+Tet). The respective mRNAs were detected by specific DNA probes, while ethidiumbromide-stained rRNAs (EtBr) serve as loading controls. Error bars correspond to standard deviation of three independent replicates. B) BN-PAGE analysis of the TIM complex in RNAi cell lines ablating either TbTim11, TbTim12 or TbTim13. Crude mitochondrial fractions were prepared after 0–4 days of RNAi induction and separated on a 4–13% BN PAGE. The TIM core component TbTim17 was in situ HA-tagged in the background of the respective RNAi cell line and detected by anti-HA antibodies. The Coomassie-stained gel serves as a loading control (Coom.). Numbers at the bottom indicate the percentage of TbTim17-HA present in the high molecular weight TIM complex. C) Immunoblots depicting steady state levels of TbTim17, TimRhom I and CoxIV in whole cell extracts of the same RNAi cell cultures as in (A). Precursor (p) and mature (m) variants of CoxIV are marked. The cytosolic protein EF1a serves as a loading control. Time of induction is indicated at the top. The graphs at the bottom show a quantification of the TbTim17 levels relative to EF1a from three to four independent experiments. The levels in uninduced cells were set to 1. Mean and standard errors of the means are indicated.

In order to examine the function of TbTim11, TbTim12 and TbTim13, we produced tetracycline-inducible RNAi cell lines. Fig 5A shows that all three proteins, like Tim8-13 analyzed in a previous study [11], are essential for normal growth of procyclic trypanosomes. Moreover, the TIM complex in these RNAi cell lines rapidly disappeared as shown by BN-PAGE (Fig 5B). However, in the same cells the mitochondrial morphology looked normal and the membrane potential was still intact till the onset of the growth phenotype (S3 Fig). Finally, in agreement with the loss of the TIM complex we observed an accumulation of unprocessed CoxIV precursors in all three small Tim RNAi-cell lines (Fig 5C), which is a hallmark of mitochondrial protein import defects.

TbTim17 belongs to the Tim17/22/23 protein family, which in yeast and humans requires small Tim chaperones for its import into mitochondria [24, 31–33]. In line with this, the steady state levels of TbTim17 are strongly decreased upon ablation of RNAi against TbTim11 and TbTim12 suggesting that the two proteins are involved in import of TbTim17. In absence of TbTim13, however, the steady levels of TbTim17 remain essentially constant (Fig 5C). The abundance of TimRhom I, another subunit of the TIM complex, is slightly reduced in the TbTim11-RNAi cell line although not to the same extent than TbTim17. In the TbTim12 and TbTim13 RNAi cell lines on the other hand the levels of TimRhom I remain essentially unchanged indicating that the protein is stable in the absence of the TIM complex.

**Fig 6:**
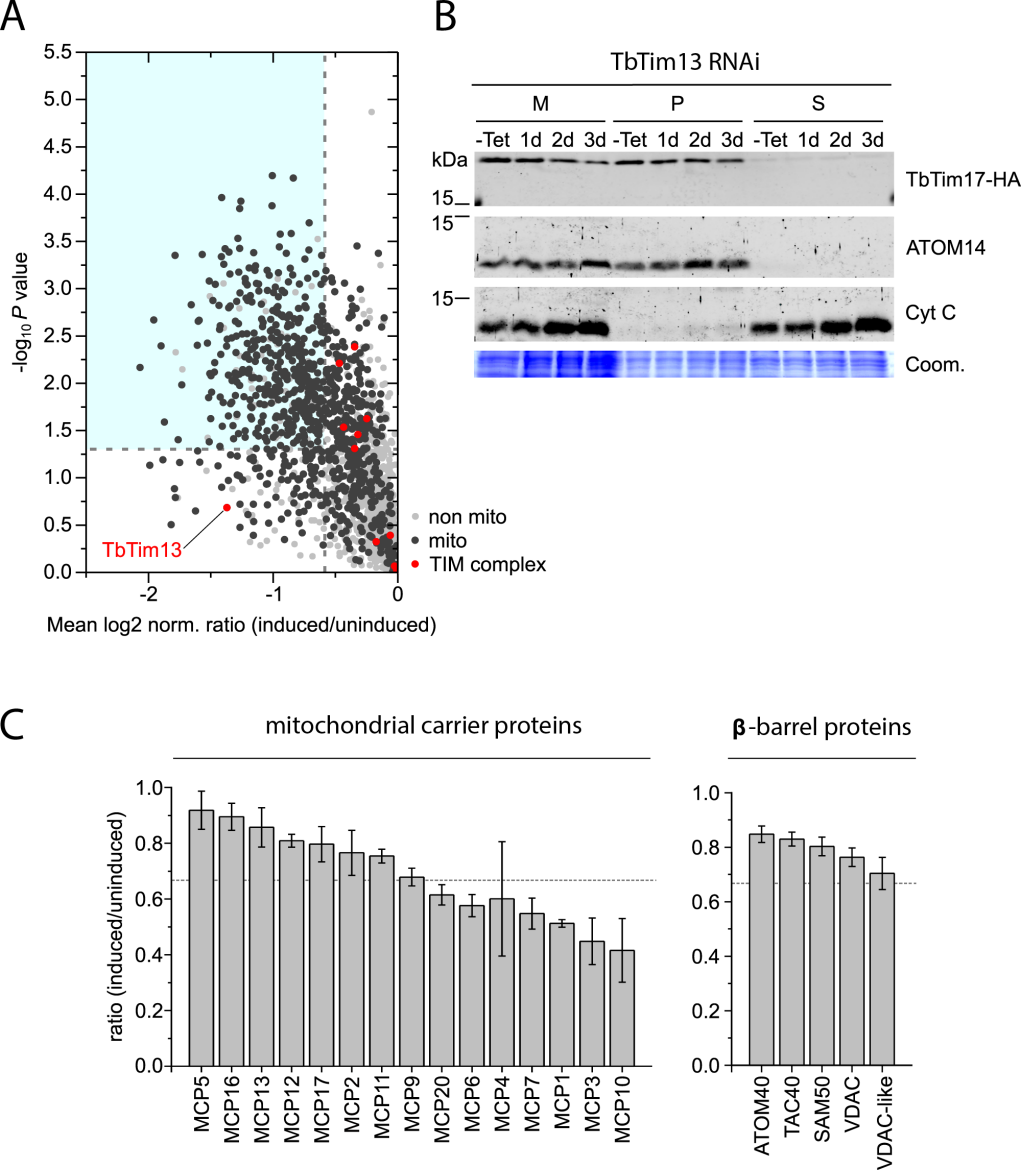
Global analysis of mitochondrial protein abundance changes upon ablation of TbTim13. A) Crude mitochondrial fractions of uninduced and induced (2.5 days) TbTim13 RNAi cells were subjected to quantitative MS using peptide stable isotope dimethyl labeling. For proteins exhibiting decreased abundance upon expression of TbTim13 RNAi, the mean log_2_ of normalized ratios (induced/uninduced) was plotted against the corresponding–log_10_ P value (two-sided t-test). The t-test significance level of 0.05 is indicated by a horizontal dashed line, while the vertical dashed line indicates a fold-reduction in protein abundance of 1.5. Mitochondrial proteins (dark grey) and TIM complex components (red) are highlighted. For a complete list of proteins see S3 Table. B) Alkaline carbonate extraction of crude mitochondrial extracts from a cell line expressing TbTim17-HA in the background of TbTim13 RNAi was performed after 0–3 days of induction. Equal cell equivalents of crude mitochondria (M), membrane pellets (P) and soluble fractions (S) were subjected to SDS-PAGE and western blotting. The single-spanning outer membrane protein ATOM14 and the IMS protein Cyt C serve as markers for the membrane and soluble fraction, respectively. The Coomassie-stained gel serves as a loading control (Coom.). C) Individual abundance ratios (induced/uninduced) of all MCPs and β-barrel proteins detected in the experiment shown in (A). Dashed horizontal line, 1.5 fold reduction. Standard deviations are indicated.

In order to investigate the function of TbTim13 in more detail we analyzed the global changes in the mitochondrial proteome that were caused by its ablation. A quantitative MS analysis of protein levels in crude mitochondrial fractions of induced versus uninduced TbTim13 RNAi cells demonstrated significantly reduced levels (≥1.5 fold, p-value ≤ 0.05) of 443 proteins (S3 Table). Most of these (86%) were mitochondrial proteins as defined in a recent proteomic study [34]. While the TIM complex is not detectable anymore after only 2 days of induction of TbTim13 RNAi (Fig 5B, right panel), the levels of the TbTim17 and all other 10 TIM complex subunits did not decrease even after 2.5 days of RNAi induction, which is when the proteomic analysis was performed (Fig 6A)[3].

For the core subunit TbTim17, alkaline carbonate extraction confirmed that the protein is still inserted into the inner membrane in the absence of TbTim13 (Fig 6B). Thus, these results strongly suggest that TbTim13, in contrast to TbTim11 and TbTim12, is directly required for the assembly and/or maintenance of the trypanosomal TIM complex but not for import of its subunits. This also explains, why its ablation essentially phenocopies the effects seen after ablation of TbTim17 [10].

We furthermore investigated the fate of two groups of proteins which are typical substrates of small Tim chaperones in other organisms, namely MCPs and β-barrel proteins. Almost half of the detected MCPs [35] were found to be significantly decreased in the TbTim13 RNAi cell line (Fig 6C). However, it is not possible to distinguish whether this phenotype is caused by impairment of the predicted chaperone function of TbTim13 or due to the more direct role it plays in TIM complex assembly and/or maintenance.

The biogenesis of β-barrel proteins like ATOM40 or VDAC, on the other hand, is independent of TbTim17 and thus the slightly reduced abundance we observed for all trypanosomal β-barrel proteins could be a direct effect of TbTim13 knockdown (Fig 6C). This is consistent with the idea that TbTim13 may facilitate the transfer of β-barrel protein across the IMS to the SAM complex. However, the observed reductions are too small to be reflected in the amounts of assembled ATOM40 and VDAC that are detected on the BN-PAGE analysis of the TbTim13 RNAi cell line (S4 Fig). The same was observed for the RNAi cell lines targeting TbTim11 and TbTim12.

## Discussion

The small Tim family of IMS-localized chaperones belongs to the most conserved components of the mitochondrial protein import system. They are found in all mitochondria-containing eukaryotes, even in trypanosomes which contain highly diverged OM and IM protein translocases [3, 4]. Here we show that the small Tim protein family in *T*. *brucei* includes six members, all of which are implicated in mitochondrial protein import [10, 11]. One of them, TbTim12, is unusual since it has an incomplete Cx3C small Tim signature motif and thus can only be stabilized by a single intramolecular disulfide bond. Nevertheless, TbTim12 clearly belongs to the small Tim family since its ablation causes the same defects that are observed in knockdown cell lines targeting classical small Tims [11]. Moreover, TbTim12 is conserved throughout kinetoplastids [12]. Thus, it is possible that similar, unusual small Tims might exist in other organisms but have escaped detection precisely because they lack complete small Tims signature domains. Also, TbTim11 and TbTim13 were not identified in previous bioinformatic analyses, even though they contain the expected Cx3C motifs. The probable reason in this case is that their Cx3C motifs are spaced by more than 16 residues [11, 12].

The trypanosomal small Tims also differ from other members of this protein family regarding the complexes they form. All six trypanosomal small Tims were found to associate with the single trypanosomal TIM complex, irrespective of whether it is engaged in MCP import or in import of presequence-containing proteins [10]. Since presequence-containing proteins generally are less hydrophobic than MCPs or other small Tim substrates, they do not rely on chaperones for the passage of the aqueous IMS. In line with this, it was not possible to show an interaction between small Tims of yeast or human and the respective presequence translocase, the TIM23 complex, even when using highly sensitive SILAC-based quantitative MS of co-immunoprecipitations [36–38]. Thus, the association of all six small Tims with the single trypanosomal TIM complex that is in the process of translocating presequence-containing substrates is very unusual.

Besides the TIM complex-associated fraction of small Tims, we demonstrated that all small Tims analyzed in our study (Tim9, TbTim11, TbTim12 and TbTim13) are also present in soluble complexes of approximately 70 kDa in mass. This suggests that trypanosomal small Tims form soluble hetero-hexameric assemblies just as small Tims in other organisms [20, 21, 23, 24]. However, the fact that all three SILAC pulldown experiments recover all six members of the small Tim protein family indicates that the postulated hexamers, unlike the ones in yeast and human, are not composed of specific pairs of small Tims. Moreover, in all cases tested, ablation of specific small Tim proteins did not result in the disappearance of the soluble, putative hexameric complexes containing other small Tim subunits. These results are most easily explained by the existence of multiple heterogeneous complexes of highly variable small Tim compositions. Thus, regarding hexamer formation, the small Tims may have at least in part redundant functions. The fact that at least four out of the six trypanosomal small Tims are individually essential for normal growth suggests that their essential function is not linked to the soluble complexes they form but to their tight association with the membrane integrated TIM translocase.

Most organisms contain a whole suite of small Tim proteins. *S*. *cerevisiae* has five such proteins, whereas humans and trypanosomes have six members of this protein family [11, 23]. The parasitic apicomplexan *Cryptosporidium* is an interesting case. It has mitosomes that lack organellar DNA, are not able to perform oxidative phosphorylation and have a highly reduced proteome. A bioinformatic analysis shows that it underwent reductive evolution resulting in a rudimentary mitochondrial protein import system which contains a single small Tim only [39]. Interestingly, this protein was able to form homo-hexameric assemblies. Moreover, even when imported into the IMS of yeast, where it would have the opportunity to interact with endogenous yeast small Tims, it only assembled with itself [39]. Thus, there appear to be three types of small Tim hexamers in nature: i) the most simple one found in *Cryptosporidium* consisting of a single small Tim subunit only, ii) the standard hexamers formed by two different small Tims arranged in alternating order in yeast and mammals and iii) the small Tim hexamers of trypanosomes which likely have highly variable subunit compositions. This suggests that while the subunits of the soluble IMS chaperone complexes all belong to the conserved small Tim protein family, the quaternary structure formed by them is quite variable in different species.

We functionally analyzed TbTim11, TbTim12 and TbTim13 by RNAi-mediated knockdown. In general, all three cell lines were found to phenocopy the mitochondrial defects observed in TbTim17 RNAi. This is explained by the fact that ablation of any of the three small Tims caused the rapid disappearance of the TIM complex. In the case of TbTim11 and TbTim12 we could demonstrate that the two proteins are required for import of TbTim17, a core component of the TIM complex. This is in agreement with results from yeast and human mitochondria where an involvement of small Tims in import of members of the Tim17/22/23 has repeatedly been shown [15, 24, 31–33]. Ablation of TbTim13 however does not affect import of TbTim17 or of other integral membrane subunits of the TIM complex, indicating that it plays a direct role in the assembly and/or maintenance of the TIM complex. This is a non-canonical function of small Tim proteins that has not been reported before.

In summary, while the number and primary structure of trypanosomal small Tims—except for TbTim12 which lacks a complete twin Cx3C motif—are similar to small Tims of other organisms, the quaternary structure of their complexes is very different. Moreover, at least one trypanosomal small Tim has a non-canonical function in TIM complex biogenesis, emphasizing the need to study this important protein family in non-standard model systems that are not closely related to yeast and mammals.

## Materials and methods

### Transgenic cell lines

Transgenic procyclic cell lines are based on *T*. *brucei* 29–13 [40] and were grown at 27°C in SDM-79 supplemented with 10% (v/v) fetal calf serum (FCS). C-terminal epitope tagging was done by fusing the full length open reading frames (ORFs) of TbTim11 (Tb927.5.3340), TbTim12 (Tb927.4.3430) and TbTim13 (Tb927.10.11520) (numbers appended to TbTim correspond to molecular weight) to C-terminal triple c-myc- or HA-tags. The fragments encoding the tagged proteins were inserted into modified pLew100 vectors [40] in which the phleomycin resistance gene had been replaced by either the puromycin or the blasticidin resistance gene [41]. RNAi constructs were prepared using stem-loop inserts, the loop being a 439 bp spacer fragment that were integrated into the same pLew100 vectors described above [42]. For TbTim11, TbTim12 and TbTim13 RNAi cell lines targeting the respective 3’UTRs were established using the primers described in S4 Table. Knockdown of TbERV1 (Tb927.9.6060) was achieved by RNAi against the ORF (nt 125–562). TbTim17 was HA-tagged in situ according to published procedures [41] in the background of different RNAi cell lines.

### Antibodies

Polyclonal rabbit antiserum targeting TbTim12 was produced using the following peptide antigen: TbTim12, aa 92–109 (EKARVEMMTQQARKELSR). For Western blots (WB) the TbTim12 antiserum was used at 1:100 dilution. The specificity was confirmed using WB of whole cell extracts of the uninduced and induced RNAi cell line. Commercially available antibodies were: mouse anti-c-myc (Invitrogen, Product No. 132500; dilution WB 1:2,000); mouse anti-HA (Enzo Life Sciences AG, Product No. CO-MMS-101 R-1000, dilution WB 1:5,000); mouse anti-EF1a (Merck Millipore, Product No. 05–235, dilution WB 1:10,000). Antibodies previously produced in our lab are: polyclonal rabbit anti-VDAC (dilution WB 1:1,000); polyclonal rabbit anti-ATOM40 (dilution WB 1:10,000, IF 1:1,000); polyclonal rabbit anti-CoxIV (dilution WB 1:1,000); polyclonal rabbit anti-Cyt C (dilution WB 1:100) and polyclonal rabbit anti-Tim9 (dilution WB 1:20); polyclonal rat anti-TbTim17 (dilution WB 1:150) [8, 10, 43]. Secondary antibodies used: goat anti-rat IRDye 680LT conjugated (LI-COR Biosciences, P/N 925–68029, dilution WB 1:10,000); goat anti-mouse IRDye 680LT conjugated (LI-COR Biosciences, P/N 926–68020, dilution WB 1:20,000); goat anti-Rabbit IRDye 800CW conjugated (LI-COR Biosciences, P/N 926–32211, dilution WB 1:20,000); goat anti-rabbit FITC conjugated (Sigma, P/N F0382, dilution IF 1:100). Immunoblots of BN-PAGEs and the tagged small Tims in 2D BN-PAGEs were decorated by HRP-coupled goat anti mouse (Sigma) as secondary antibodies, dilution 1:5000.

### Digitonin extraction

To generate crude mitochondria enriched fractions by selective lysis of the plasma membrane [44], 1∙10^8^ cells were incubated for 10 min on ice in 20 mM Tris-HCl pH 7.5, 0.6 M sorbitol, 2 mM EDTA containing 0.015% (w/v) digitonin. After centrifugation (6,800 g, 4°C), the resulting mitochondria enriched pellet was separated from the supernatant and subjected to SDS-PAGE (2.5 10^6^ cell equivalents of each fraction) and immunoblotting to demonstrate mitochondrial localization of a protein of interest. Alternatively, the mitochondria enriched pellet was used for further experiments. For visualization of tagged proteins, the respective cell lines were induced for 24 h with tetracycline.

### Submitochondrial fractionation by digitonin

Crude mitochondrial fractions produced as described above were further incubated with 0.2% digitonin (20 mM Tris-HCl pH 7.5, 0.6 M sorbitol, 2 mM EDTA) for 15 min on ice to selectively open the outer mitochondrial membrane. By centrifugation (6,800 g, 4°C) the supernatant containing released IMS proteins could be separated from the membrane fraction in the pellet. The individual fractions were subjected to further experiments.

### Alkaline carbonate extraction

To separate soluble proteins from membrane-attached ones, a mitochondria enriched pellet fraction obtained by digitonin extraction was resuspended in 100 mM Na_2_CO_3_ at either pH 11.5 or pH 10.7, incubated on ice for 10 min and centrifuged (100,000 g, 4°C, 10 min). Equal cell equivalents of all samples were analyzed by SDS-PAGE und immunoblotting.

### Co-immunoprecipitation

For co-immunoprecipitations of tagged TbTim11, TbTim12 and TbTim13, digitonin-extracted crude mitochondrial fractions or submitochondrial fractions of 1∙10^8^ cells each were solubilized for 15 min at 4°C in 20 mM Tris-HCl, pH 7.4, 0.1 mM EDTA, 100 mM NaCl, 10% glycerol containing 1% (w/v) digitonin and 1X Protease Inhibitor mix (EDTA-free, Roche). Following a clearing spin (20,000 g, 15 min, 4°C), the lysate (load) was transferred to affinity purification resin (30 μl EZview red anti-c-myc affinity gel from Sigma or 50 μl anti-HA affinity matrix from Roche) that had been equilibrated in wash buffer (20 mM Tris-HCl, pH 7.4, 0.1 mM EDTA, 100 mM NaCl, 10% glycerol containing 0.2% (w/v) digitonin). After 2 h of incubation at 4°C, the supernatant (unbound proteins) was removed and the resin was washed 3 times with 500 μl wash buffer. To elute the bound proteins, the resin was boiled for 5 min in 2% SDS in 60 mM Tris-HCl pH 6.8 (eluate). The resulting samples were analyzed by SDS-PAGE and Western blotting.

### SILAC Co-immunoprecipitation

SILAC co-immunoprecipitation experiments were essentially done as described before [10]. *T*. *brucei* cell lines allowing inducible expression of C-terminally HA-tagged TbTim11, TbTim12 and TbTim13 were used. Expression of the tagged small Tim proteins was induced for 1 d. Cells were grown in SDM-80 medium containing either ^12^C_6_^14^N_x_ ("light") or ^13^C_6_^15^N_x_ ("heavy") arginine and lysine. Digitonin-extracted IMS-containing fractions were processed for immunoprecipitation as described above (see "Co-immunoprecipitation"). Bound proteins were eluted by boiling the resin for 5 min in 60 mM Tris HCl, pH 6.8, containing 0.1% SDS and separated by SDS-PAGE on a 4–12% NuPAGE BisTris gradient gel (Life Technologies). Proteins were stained using colloidal Coomassie Brilliant Blue and gel lanes cut into 10 slices. For each bait protein, three biological replicates including a label-switch were performed. Sample preparation including reduction, alkylation, and tryptic in-gel digestion of proteins as well as LC-MS measurements using an Orbitrap Elite mass spectrometer and quantitative MS data analysis with MaxQuant/Andromeda (version 1.5.5.1 [45];[46]) were performed as described before [10]. The mean log_10_ of protein abundance ratios (TbTim11/12/13-HA versus control) and the p-value (one-sided Student's t-test) across ≥ 2 biological replicates were determined. See S2 Table for information about proteins identified (overall sequence coverage ≥ 4%) and quantified in SILAC co-immunoprecipitation experiments.

### Immunofluorescence microscopy

For analysis of the mitochondrial membrane potential, uninduced and induced TbTim11, TbTim12 and TbTim13 RNAi cell lines were grown for 15 min in the presence of 250 nM MitoTracker Red CMXRos. Cells were harvested, washed with PBS, fixed with 4% paraformaldehyde in PBS and permeabilized with 0.2% Triton X-100 in PBS. Between the different incubation steps with primary and secondary antibodies, cells were repeatedly washed with PBS. After postfixation in cold methanol, the slides were mounted using VectaShield containing 4',6-diamidino-2-phenylindole (DAPI) (Vector Laboratories, P/N H-1200). Images were acquired with a DFC360 FX monochrome camera (Leica Microsystrems) mounted on a DMI6000B microscope (Leica Microsystems). Image analysis was done using LAS X software (Leica Microsystems).

### RNA extraction and Northern blotting

Isolation of total RNA from uninduced and induced (2 days) RNAi cells was performed using acid guanidinium thiocyanate-phenol-chloroform extraction [47]. RNA was separated on a 1% agarose gel in 20 mM MOPS buffer, pH7.0 containing 0.5% formaldehyde and transferred to a Nylon membrane (GeneScreen Plus, PerkinElmer) by passive diffusion. Northern probes were prepared from gel-purified PCR products corresponding to the RNAi inserts described above and radioactively labelled using the Prime-a-Gene labelling system (Promega).

### BN-PAGE and 2D BN-PAGE

Mitochondria enriched fractions or submitochondrial fractions were used as starting material and solubilized in 20 mM Tris-HCl pH 7.4, 50 mM NaCl, 10% glycerol, 0.1 mM EDTA containing 1% (w/v) digitonin. The lysates were cleared by centrifugation prior to separation on 4–13% gradient gels for 1D BN-PAGE of the TIM complex and 6–16.5% BN-PAGE for 2D PAGE. For the 2D PAGE the protein complex containing lane was excised, incubated in SDS sample buffer for 15 min at room temperature, boiled for 20 s and again incubated for 20 min at room temperature before the second dimension separation on a SDS-PAGE (14%). To facilitate transfer of proteins to membranes, BN-PAGE and 2D BN-PAGE gels were incubated in SDS-PAGE running buffer (25 mM Tris, 1 mM EDTA, 190 mM glycine, 0,05% (w/v) SDS) prior to Western blotting.

### Quantitative mass spectrometry-based analysis of TbTim13 RNAi cells

Mitochondria enriched fractions from induced and uninduced TbTim13 RNAi cells were resuspended in 8 M urea/50 mM NH_4_HCO_3_ and processed for liquid chromatography-mass spectrometry (LC-MS) analysis as described previously [34] with slight modifications. In brief, proteins were reduced, alkylated, and tryptically digested followed by differential stable isotope dimethyl labeling [48] of desalted peptides using "light" formaldehyde (CH_2_O) and sodium cyanoborohydride (NaBH_3_CN) or the "heavy", deuterated counterparts (CD_2_O/NaBD_3_CN). The experiment was performed in biological triplicates including a label-switch. Light and heavy dimethyl-labeled peptides each derived from 10 μg of protein of mitochondrial fractions from induced and uninduced TbTim13 RNAi cells were mixed, purified using StageTips, and fractionated by high pH reversed-phase (RP) chromatography. To this end, acidified peptide mixtures were loaded onto StageTips, washed twice with 0.5% (v/v) acetic acid and eluted step-wise with 0%, 3%, 6%, 10%, 13%, 15.6%, 18.7%, and 72% (v/v each) acetonitrile in 10 mM NH_4_OH. LC-MS analyses, performed using an Orbitrap Elite (Thermo Fisher Scientific, Bremen, Germany) coupled to an UltiMate 3000 RSLCnano HPLC system (Thermo Fisher Scientific, Dreieich, Germany), and subsequent MS data analysis using the MaxQuant/Andromeda software platform (version 1.5.5.1 [45];[46]) with parameters specific for stable dimethyl labeling were carried out as described by [34]. The mean log_2_ of normalized protein abundance ratios (induced/uninduced) and the p-value (two-sided Student's t-test) across ≥ 2 biological replicates were determined. See S3 Table for information about proteins identified (overall sequence coverage ≥ 4%) and quantified in this analysis.

## Supporting information

S1 Fig Characterization of Erv1-RNAi cell lines. Growth curve and corresponding Northern blots of uninduced and induced ERV1-RNAi cell lines allowing inducible expression of TbTim11-HA (left graph), TbTim12-HA (middle graph) and TbTim13-HA (right graph), respectively. Standard deviations are indicated.
(EPS)

S2 Fig Control blots for the bottom two panels of Fig 3A and 3B. A) 2D BN-PAGE analyses of crude mitos from cell lines expressing either TbTim11-HA or TbTim13-HA were probed for TbTim42 (controls for Fig 3A bottom two panels). The position of TbTim17-myc which was mixed into the sample is also indicated. B) 2D BN-PAGE analyses of soluble and membrane fractions from cell lines expressing either TbTim11-HA of TbTim13-HA were probed for TbTim17, TbTim42 and CytC (controls for Fig 3B, bottom two panels). The gels were aligned to the 66 kDa marker. The panels depicting TbTim12-HA and TbTim13-HA for (A) and (B) are identical to the ones shown in Fig 3A and 3B of the main manuscript.
(EPS)

S3 Fig Mitochondrial morphology and membrane potential are not affected by RNAi-mediated ablation of TbTim11, TbTim12 or TbTim13 until the onset of the growth phenotype. Immunofluorescence microscopy of RNAi cell lines induced for 0–3 days. Membrane potential was detected with Mitotracker. Differential interference contrast (DIC) and DAPI staining of DNA depict general cell morphology. ATOM40 serves as mitochondrial marker.
(TIF)

S4 Fig β-barrel protein containing complexes are not affected by RNAi-mediated ablation of individual small Tims. A) BN-PAGE analysis of the ATOM complex in RNAi cell lines ablating either TbTim11, TbTim12 or TbTim13. Crude mitochondrial fractions were prepared after 0–4 days of RNAi induction and separated on a 4–13% BN PAGE. Anti-ATOM40 antibody was used to detect the β-barrel core subunit ATOM40. The Coomassie-stained gel serves as a loading control. B) BN-PAGE analysis of the β-barrel protein VDAC in RNAi cell lines ablating either TbTim11, TbTim12 or TbTim13 was performed as in (A).
(EPS)

S1 Table Sequence comparison of trypanosomal small Tims with *S*. *cerevisiae* and human small Tims. Sequence similarity and identity of the respective protein pairs was analyzed by the SIAS (Sequence Identity And Similarity) tool by Reche, P. (2008) (available at http://imed.med.ucm.es/Tools/sias.html).
(XLSX)

S2 Table Lists of proteins detected in SILAC co-immunoprecipitation experiments of small Tim proteins. A) Proteins identified (overall sequence coverage ≥ 4%) in TbTim11-HA pull down experiments. B) Proteins identified (overall sequence coverage ≥ 4%) in TbTim12-HA pull down experiments. C) Proteins identified (overall sequence coverage ≥ 4%) in TbTim13-HA pull down experiments.
(XLSX)

S3 Table Proteins detected in quantitative MS analyses of TbTim13 RNAi cells. Listed are all proteins identified with an overall sequence coverage of ≥ 4%.
(XLSX)

S4 Table Primers used for cloning. (XLSX)
