# Supplementary figures and images for: A trypanosomal orthologue of an intermembrane space chaperone has a non-canonical function in biogenesis of the single mitochondrial inner membrane protein translocase
Source: PLoS Pathog. 2017 Aug 21;13(8):e1006550. doi: 10.1371/journal.ppat.1006550 (PMC5584982; doi:10.1371/journal.ppat.1006550)

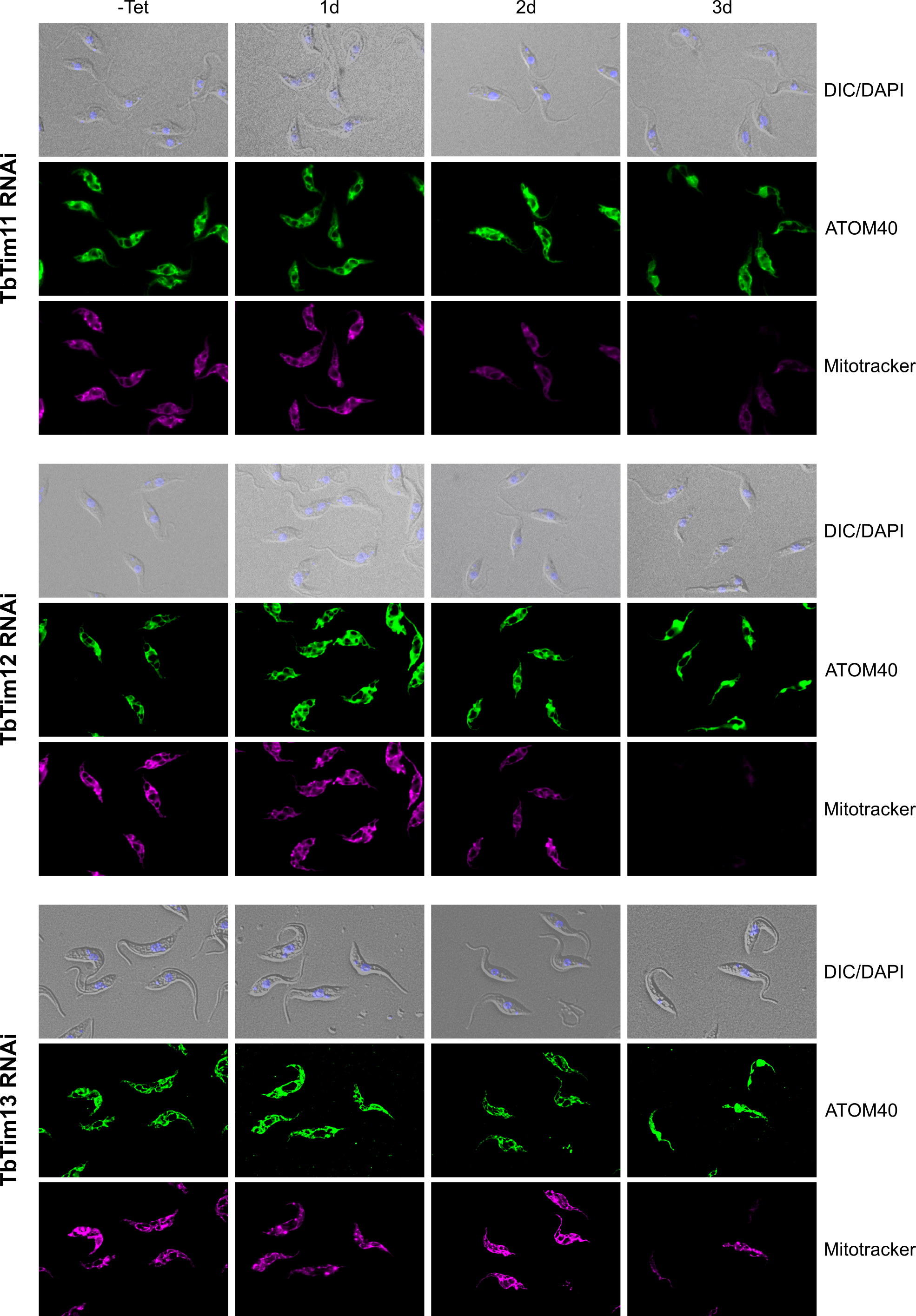

Supplement: S3 Fig — Immunofluorescence microscopy of RNAi cell lines induced for 0–3 days. Membrane potential was detected with Mitotracker. Differential interference contrast (DIC) and DAPI staining of DNA depict general cell morphology. ATOM40 serves as mitochondrial marker. (TIF) [file ppat.1006550.s003.tif]
